# Influence of thread design on anchorage of pedicle screws in cancellous bone: an experimental and analytical analysis

**DOI:** 10.1038/s41598-022-11824-2

**Published:** 2022-05-16

**Authors:** Martin Weidling, Martin Heilemann, Stephan Schoenfelder, Christoph E. Heyde

**Affiliations:** 1 Department of Orthopaedic Surgery, Traumatology and Plastic Surgery, ZESBO - Center for Research on Musculoskeletal Systems, Leipzig University, Leipzig, Germany; 2 Faculty of Engineering, University of Applied Sciences Leipzig, Leipzig, Germany; 3 Department of Orthopaedic Surgery, Traumatology and Plastic Surgery, Leipzig University Medical Center, Leipzig, Germany

**Keywords:** Mechanical engineering, Bone, Fracture repair, Orthopaedics

## Abstract

Threads of modern pedicle screws can vary greatly in design. It is difficult to assess which interplay of design features is particularly advantageous for screw anchorage. This study aims to increase the understanding of the anchorage behaviour between screw and cancellous bone. Pull-out tests of six pedicle screws in two sizes each were performed on three densities of biomechanical test material. More general screw characteristics were derived from the screw design and evaluated using the test data. Selected screws were tested on body donor material. Some screw characteristics, such as compacting, are well suited to compare the different thread designs of screws with tapered core. The combination of two characteristics, one representing bone compacting and one representing thread flank area, appears to be particularly advantageous for assessing anchorage behaviour. With an equation derived from these characteristics, the pull-out strength could be calculated very accurately (mean deviation 1%). Furthermore, findings are corroborated by tests on donor material. For screws with tapered core, the design demands for good anchorage against pull-out from cancellous bone change with material density. With sufficient bone quality, screws with a high compacting effect are advantageous, while with low bone density a high thread flank area also appears necessary for better screw anchorage.

## Introduction

Screw diameter, screw length, screw design, and bone quality are often mentioned in the literature as the most important factors influencing pedicle screw anchorage^1–7^. However, the choice of screw diameter and screw length strongly depends on the individual anatomy and surgical technique^8–10^. In addition, the bone quality is decisively influenced by the patient's state of health and age^11^. Thread design, on the other hand, can be adapted and is therefore the subject of research and development. Various design features of screws and their influence on anchorage have already been studied^12–14^. In some cases, screws have been manufactured specifically for the purpose of comparison, differing only in one design feature^15–17^. However, which combination of features provides the best anchorage is not yet fully understood. As a result, the screws used in clinical practice have very different thread designs.

One approach is to look at more general screw characteristics, taking into account several design features. These may allow statements about the achievable anchoring effect. For example, screw characteristics such as flank overlap area, contact area, bone compacting or insertion torque have been suggested^18–23^. Often this is conjecture, or there is little data available. There is a lack of comprehensive studies to further assess the relevance of these characteristics. Furthermore, it is assumed that the compacting of the bone together with the thread flank area is decisive for the anchorage of screws, but corresponding studies are lacking^24^. An analytical equation that takes into account the screw diameter, screw length, screw design and bone quality appears valuable for screw development and evaluation of the anchorage effect. Chapman et al.^14^ have proposed an equation for screw pull-out here. However, this has weaknesses as it does not include the effects of pre-drilling or tapping or the compaction effect of modern screws^25^.

The aim of this work is to broaden the understanding of the anchorage behaviour between screws and cancellous bone. To this end, examinations are carried out in four steps. First, six types of pedicle screws, each in two sizes, are tested in pull-out tests on three densities of synthetic foam, and the measurement results are compared. Secondly, it is examined whether more general screw characteristics are suitable for assessing screw anchorage. Thirdly, it is analytically evaluated whether the combination of two parameters, one describing the compacting of bone and one describing the thread flank area, allows statements about screw anchorage. For this purpose, an equation for calculating the pull-out strength from these parameters is established. The results are compared with the measurement results and Chapman's equation. Finally, the findings obtained on synthetic material are verified on a more complex material. For this purpose, pull-out experiments are performed on cancellous bone from human donor material in order to classify the results quantitatively.

## Methods

### Experimental testing

**Figure 1:**
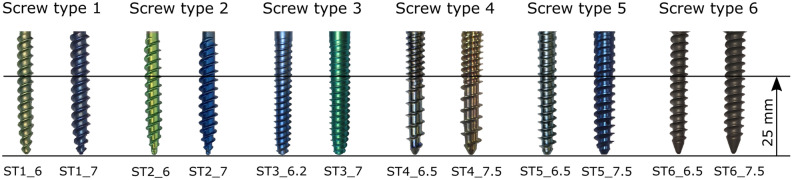
Six screw types examined, each in two dimensions, with the thread range considered from the tip to 25 mm.

Six screw types were selected, each with two different outer diameters. All of them were approved pedicle screws in clinical use, with the exception of the screw type 5, which was a prototype (Königsee Implantate GmbH, Germany). The screws were made of titanium alloy and 45 mm long. The thread region of all screws is shown in Fig. 1. The outer diameter of the screws varied from 6 to 7.5 mm. All screws had a tapered core diameter, except type 6, which had a cylindrical core diameter. Thread pitch and core diameter were determined with a calliper gauge (Table 2). For screws with tapered core, the mean core diameter was determined by measuring each thread turn of the first 25 mm.

Polyurethane foam (Sawbones, Pacific Research Laboratories Inc., USA) according to ASTM F1839^26^ in grades PCF 15 (0.24 g/cm^3^), PCF 10 (0.16 g/cm^3^), and PCF 5 (0.08 g/cm^3^) was used as homogeneous test material. Foams with apparent densities of 0.24 g/cm^3^ were used as a model for normal, 0.16 g/cm^3^ for osteoporotic and 0.08 g/cm^3^ for severely osteoporotic bone^5^. The densities of each group may vary slightly (up to ± 10% according to the manufacturer). To obtain reproducible results, recommendations for sample preparation were followed^27^. The apparent density was determined for each foam block according to ASTM D1622^28^. Foam blocks with as equal a density as possible were selected. Next to this, the blocks were divided so that each screw was tested in each foam block. In addition, the foam blocks were pre-drilled perpendicular to the surface to a diameter of 2.5 mm up to a length of 28 mm.

**Figure 2:**
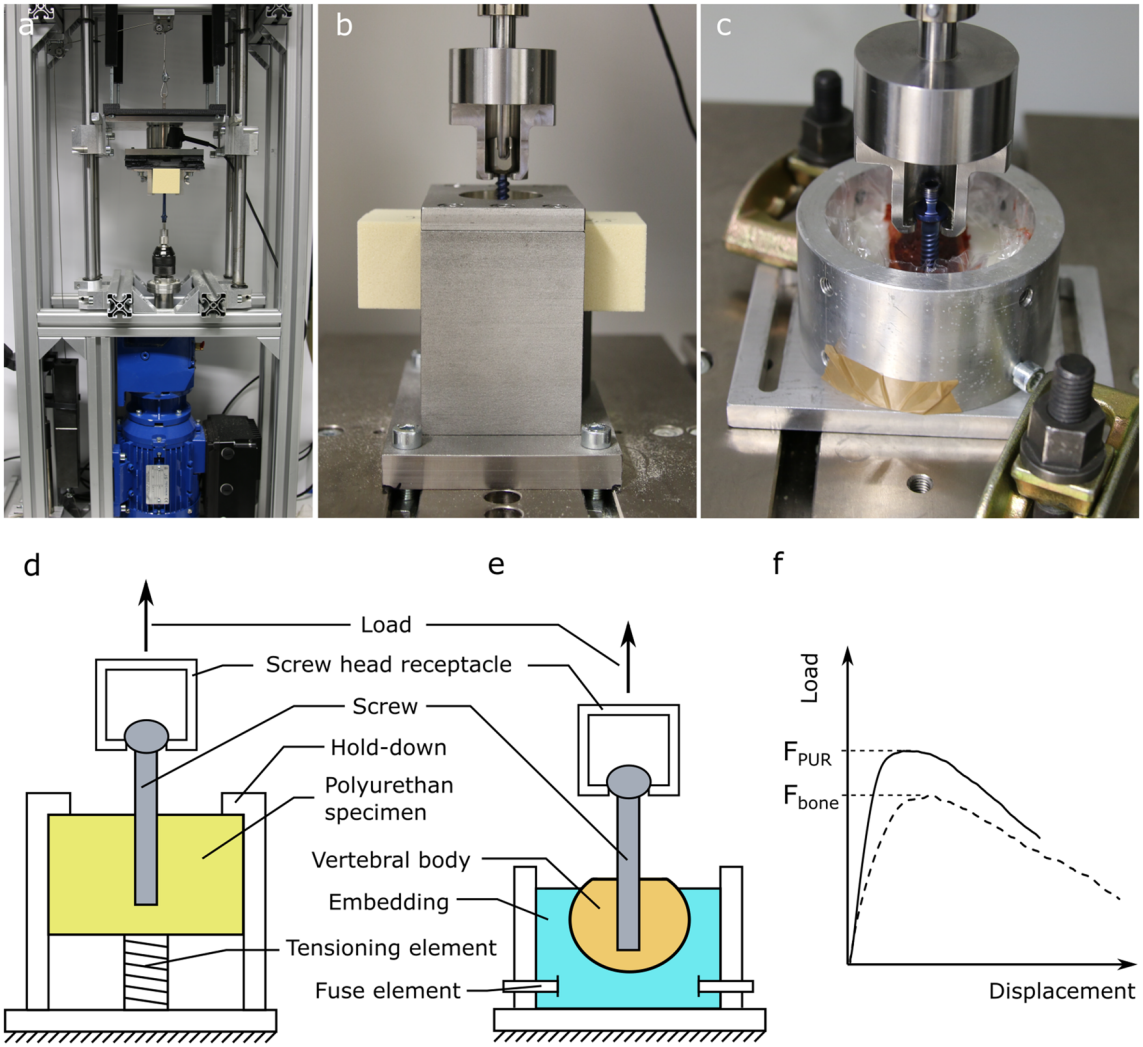
Experimental set-up: (**a**) screw-in test rig, (**b**) pull-out test from synthetic foam, (**c**) pull-out test from vertebral body of a body donor, (**d**) pull-out test from synthetic foam schematic, (**e**) pull-out test from vertebral body of body donor schematic and (**f**) qualitative progression of representative force–displacement curves from polyurethane foam and bone pull-out.

All screws were inserted perpendicularly into the foam blocks by machine on a self-developed screw-in test rig based on ASTM F543^29^, cf. Fig. 2a. At a speed of 3 rpm the screws were screwed into the foam block by 25 mm. Along the first 10 mm of the screw-in path, a vertical force was applied to all screws until the screws were gripped in the foam. In a preliminary test, the required forces were determined to be 60 N for PCF 15, 30 N for PCF 10 and 15 N for PCF 5, with the exception of screw type 6, which required twice the force in each case. A force of 11 N was applied for the remaining screw-in distance to hold the bit in the screw. The torque was measured continuously by a 6-Axis force/torque sensor (K6D40; ME-Meßsysteme GmbH, Germany), with the highest value interpreted as the insertion torque.

Subsequently, the screws were pulled out of the foam as per ASTM F543. Accordingly, a spacing of the screws in the foam of at least five times the diameter was maintained. In addition, the opening of the hold-down was at least five times the screws’ diameter. Finally, the screws were pulled out axially and perpendicular to the foam specimen at a speed of 5 mm/min. The receptacle for the screw head caused the application of pure tensile forces and prevented bending moments, cf. Fig. 2b, d. The force–displacement curve was recorded by a material testing machine (Zwick, Germany). The pull-out strength was determined as the maximum force from the force–displacement curves, cf. Fig. 2f. Six pull-outs were performed for each screw for each foam grade, resulting in 216 tests performed.

### Screw characteristics

Eight screw characteristics (obliquely highlighted) were identified and tested for a linear relationship with the measured pull-out strength. Here, the *outer diameter* $D_{o}$ of the screw was considered^30,31^. The *flank overlap area* $FOA$ was calculated according to

1 $$ FOA = \frac{\pi }{4} \cdot \left( {D_{o}^{2} - D_{c}^{2} } \right) \cdot \frac{L}{p} $$

depending on outer diameter D_o_, mean core diameter D_c_, screw length L, and screw pitch p^18^. The *contact surface* $A_{c}$ in the screw-bone interface was determined from virtual 3D models of the screws. A quantitative measure of *compacting bone* $V_{c}$ was calculated by

2 $$ V_{c} = \frac{{V_{d} }}{{\frac{\pi }{4} D_{o}^{2} L}} $$

with V_d_ as displaced bone volume when a screw was inserted^24^. The displaced volume was obtained from virtual 3D models of the screws and corresponded to the volume of the screw part located in the bone material minus the volume of the pilot hole. Furthermore, the *displaced diameter* $d_{d}$ with

3 $$ d_{d} = 2 \cdot \sqrt {\frac{{V_{d} }}{\pi L}} $$

was considered as a measure for compacting^24^. Moreover, the *insertion torque* $T_{i}$ was taken into account^20,21^. In addition, the *product of outer diameter and insertion torque* $D_{o} \cdot T_{i}$, as well as the *product of outer diameter and compacting* $D_{o} \cdot V_{c}$ were considered^24^.

### Analytical evaluation

During further analysis, the influence of compacting together with the thread flank area on the pull-out strength was evaluated analytically. Chapman et al.^14^ proposed an equation for calculating the pull-out strength of cancellous bone screws as

4 $$ F_{Ch} = \tau_{s} \cdot A_{s} \cdot TSF = \tau_{s} \cdot \pi D_{o} L_{s} \cdot \left( {\frac{1}{2} + \frac{1}{\sqrt 3 }\frac{d}{p}} \right) $$

This takes into account the shear strength $\tau_{s}$ of the test material and the area of the shear zone $A_{s}$ (with L_s_ as the shear length). Whereby the so-called thread shape factor TSF considers the thread design over the thread depth $d = \frac{{D_{o} - D_{c} }}{2}$ and the thread pitch $p$.

In this study, the Chapman equation was built upon. The correction factor $CF$ was introduced to take into account the influence of the screw on the achievable pull-out strength in

5 $$ F_{p} = \tau_{s} \cdot \pi D_{o} L_{s} \cdot CF. $$

First, the ideal correction factor $CF_{ideal}$ was determined from the experimentally measured pull-out strength $F_{exp}$ for all screws and densities with

6 $$ CF_{ideal} = \frac{{F_{exp} }}{{\tau_{s} \cdot \pi D_{o} L_{s} }}. $$

The shear strengths $\tau_{s}$ of the polyurethane foam were taken from the manufacturer's data (cf. Table 5), and the shear lengths L_s_ were determined experimentally (cf. Appendix 1). Next, it was investigated whether the ideal correction factor is linearly related to two parameters, one of which represents compacting $P_{C}$ and one of which represents thread flank area $P_{F}$. For this purpose, several dimensionless quantities were introduced in Table 1. For each parameter combination of $P_{C}$ and $P_{F}$, a 3D regression was performed in MATLAB (The MathWorks, Inc., USA). This resulted in a linear function for the correction factor with two parameters $CF\left( {P_{C} ,P_{F} } \right)$ and corresponds to a plane equation

7 $$ CF = CF\left( {P_{C} ,P_{F} } \right) = A \cdot P_{C} + B \cdot P_{F} + C. $$

**Table 1:** Parameters considered for compacting $P_{C}$ and thread flank area $P_{F}$, with V_d_—displaced bone volume, D_o_—outer diameter, D_c_—core diameter, L—screw length, V_c_—bone compacting, p—screw pitch, d—thread depth.

Compacting parameters $P_{C}$	Thread flank area parameters $P_{F}$
Compacting between thread flanks $P_{C\_1} = \frac{{V_{d} }}{{\frac{\pi }{4} \left( {D_{o}^{2} - D_{c}^{2} } \right) L}}$	Relative flank area over thread length $P_{F\_1} = \frac{{D_{o}^{2} - D_{c}^{2} }}{{D_{o}^{2} }} \cdot \frac{L}{p}$
Compacting $P_{C\_2} = V_{c} = \frac{{V_{d} }}{{\frac{\pi }{4} D_{o}^{2} L}}$	Relative flank area per thread turn $P_{F\_2} = \frac{{D_{o}^{2} - D_{c}^{2} }}{{D_{o}^{2} }}$
Displaced diameter $P_{C\_3} = 2 \cdot \sqrt {\frac{{V_{d} }}{\pi L}} \cdot \frac{1}{\left[ m \right]}$	Ratio of thread depth to thread pitch $P_{F\_3} = \frac{d}{p}$
Outer diameter times compacting $P_{C\_4} = D_{o} \cdot V_{c} \cdot \frac{1}{\left[ m \right]}$	Number of thread turns per thread length $P_{F\_4} = \frac{L}{p}$

Based on the coefficient of determination R^2^, the best-fit parameter combination was selected, and the coefficients A, B and C of Eq. (7) were determined. This was used to calculate the correction factor $CF$ for each screw dependent on its compacting characteristic and thread flank area. Subsequently, the pull-out strengths were calculated according to Eq. (5). The results were compared with the results from the Chapman equation Eq. (4) and the experimental results for the three foam densities.

### Donor bones

All donors originated from the Institute of Anatomy of the Leipzig University and had given written consent to dedicate their bodies to medical education and research purposes. Being part of the body donor program regulated by the Saxonian Death and Funeral Act of 1994 (3rd section, paragraph 18, item 8), institutional approval for the use of the post-mortem tissues of human body donors was obtained. The authors declare that all experiments were performed according to the ethical principles of the Declaration of Helsinki.

Twelve fresh-frozen human vertebrae (th11 and th12) from six body donors [three male and female each, age: 86 years (SD 6 years)] were harvested, cf. Table 4. The bone mineral density (BMD) was determined by dual-energy X-ray absorptiometry (DEXA; Delphi A, HOLOGIC, USA) in accordance with the clinical setting. In addition, computed tomography (CT; Brilliance iCT256, Philips, The Netherlands) scans were performed, after which two vertebrae had to be excluded because of deficiencies. Specimens were stored wrapped in plastic foil at − 80 °C and then gently thawed. The vertebrae were separated, and all soft tissues were removed. The posterior wall of the vertebral body was then removed to reveal the cancellous bone.

The vertebral bodies were embedded in a ring with a polyurethane casting resin (RenCast®, Huntsman Int. LLC, USA). Two screw types (ST2_7 and ST3_6.2) were examined and each tested on one vertebral body. The test was performed analogously to the tests on synthetic foam. One screw was screwed into the vertebral body laterally on the left, the other laterally on the right. Sufficient space was left between the screws to rule out any interference. Careful planning of the drill holes and control of the screw-in path during pre-drilling ensured that the screws were inserted in a purely cancellous manner. This was followed by the axial pull-out, cf. Fig. 2c, e. The order of the screws was alternated during the experiment.

### Statistical analysis

Statistical analyses were performed with SPSS 24.0 (IBM Analytics, USA). The Shapiro–Wilk test was used to check the series of measurements for normal distribution. Differences in the means of the synthetic foam specimens were analysed with the two-tailed t-test for normally distributed data, otherwise the Mann–Whitney test was used. The measurement data from the experiments with donor bones were tested with the two-tailed paired t-test. In addition, Cohen's effect size d was determined (0.2 ≤ d < 0.5 small effect, 0.5 ≤ d < 0.8 medium effect, 0.8 ≤ d < 1 large effect)^32^. Linear regression analyses were carried out to examine the influence of different screw characteristics on the pull-out strength and the relationship between insertion torque and pull-out strength. For correlations with two parameters, a 3D regression was performed. The coefficient of determination R^2^ was determined to assess the correlation. In all statistical analysies a value *p* < 0.05 was considered statistically significant.

## Results

### Experimental testing

The measured values (mean value ± one standard deviation) are listed in Table 2. All test groups are normally distributed except the insertion torques of ST1_6 for PCF 15. For all screw types in all three foam densities, both insertion torque and pull-out strength were significantly higher for larger diameter screws than for smaller diameter screws, with the exception of pull-out strength for screw type 6. With screw type 6, the pull-out strengths of the screws with the large diameter are significantly lower in relation to the smaller ones with PCF 15, with PCF 10 there is no significant difference and with PCF 5 they are significantly higher. In addition, differences in the mean values for the insertion torque [*p* = 0.15 (PCF 15), *p* = 0.038 (PCF 10), *p* < 0.001 (PCF 5)] and the pull-out strength [*p* = 0.001 (PCF 15), *p* = 0.003 (PCF 10), *p* < 0.001 (PCF 5)] were determined for the screws ST2_7 and ST3_6.2.

**Table 2:** Screw properties and measured values (mean value ± one standard deviation) including screw characteristics: outer diameter, flank overlap area, contact surface, compacting, displaced diameter, insertion torque, $D_{o} \cdot V_{c}$ and $D_{o} \cdot T_{i}$.

Screw properties	Screw type 1^a^		Screw type 2^a^		Screw type 3^a^		Screw type 4		Screw type 5		Screw type 6	
	ST1_6	ST1_7	ST2_6	ST2_7	ST3_6.2	ST3_7	ST4_6.5	ST4_7.5	ST5_6.5	ST5_7.5	ST6_6.5	ST6_7.5
Outer diameter $D_{o}$ in mm	6	7	6	7	6.2	7	6.5	7.5	6.5	7.5	6.5	7.5
Mean core diameter $D_{c}$ in mm	3.65	4.04	3.86	3.94	4.36	5.03	4.15	4.78	4.25	4.99	4.45	5.10
Screw pitch $p$ in mm	3.00	3.00	3.00	3.00	2.00	2.00	4.00	4.00	2.75	2.75	2.80	2.80
Flank overlap area $FOA$ in mm^2^	148	214	138	219	191	232	123	164	173	224	157	212
Contact surface $A_{c}$ in mm^2^	429	532	430	519	548	599	464	564	591	716	500	605
Compacting $V_{c}$	0.32	0.35	0.31	0.28	0.41	0.45	0.34	0.34	0.41	0.45	0.37	0.41
Displaced diameter $d_{d}$ in mm	1.69	2.07	1.67	1.86	1.99	2.35	1.89	2.12	2.07	2.52	1.99	2.40
$D_{o} \cdot V_{c}$ in mm	1.90	2.45	1.86	1.98	2.56	3.15	2.19	2.55	2.63	3.37	2.43	3.06
**Measured values**												
*PCF 15 (237.2 ± 0.3) kg/m*^*3*^												
Insertion torque $T_{i}$ in Nm	1.36 ± 0.07	1.88 ± 0.07	1.31 ± 0.07	1.83 ± 0.03	1.82 ± 0.01	2.50 ± 0.03	1.32 ± 0.08	1.72 ± 0.11	1.72 ± 0.06	2.59 ± 0.02	1.20 ± 0.05	1.67 ± 0.03
Mean value difference	*p* = 0.002		*p* < 0.001		*p* < 0.001		*p* < 0.001		*p* < 0.001		*p* < 0.001	
Pull-out strength $F_{exp}$ in N	870 ± 10	931 ± 12	855 ± 12	923 ± 12	965 ± 20	1074 ± 16	902 ± 10	975 ± 18	966 ± 18	1072 ± 12	858 ± 19	798 ± 24
Mean value difference	*p* < 0.001		*p* < 0.001		*p* < 0.001		*p* < 0.001		*p* < 0.001		*p* = 0.001	
$D_{o} \cdot T_{i}$ in Nm·mm	8.29	13.23	8.00	12.83	11.27	17.57	8.58	13.03	11.31	19.41	7.71	12.55
*PCF 10 (158.0 ± 0.5) kg/m*^*3*^												
Insertion torque $T_{i}$ in Nm	0.60 ± 0.02	0.93 ± 0.03	0.60 ± 0.07	0.90 ± 0.04	0.95 ± 0.03	1.26 ± 0.05	0.63 ± 0.02	0.87 ± 0.01	0.90 ± 0.03	1.31 ± 0.05	0.57 ± 0.02	0.80 ± 0.04
Mean value difference	*p* < 0.001		*p* < 0.001		*p* < 0.001		*p* < 0.001		*p* < 0.001		*p* < 0.001	
Pull-out strength $F_{exp}$ in N	446 ± 4	476 ± 5	441 ± 13	479 ± 15	510 ± 13	555 ± 10	445 ± 12	491 ± 9	485 ± 13	534 ± 13	426 ± 6	435 ± 17
Mean value difference	*p* < 0.001		*p* = 0.001		*p* < 0.001		*p* < 0.001		*p* < 0.001		*p* = 0.25	
$D_{o} \cdot T_{i}$ in Nm·mm	3.65	6.48	3.45	6.31	5.91	8.93	4.07	6.55	5.87	9.94	3.74	5.94
*PCF 5 (85.8 ± 0.2) kg/m*^*3*^												
Insertion torque $T_{i}$ in Nm	0.22 ± 0.01	0.35 ± 0.02	0.18 ± 0.01	0.31 ± 0.01	0.38 ± 0.01	0.51 ± 0.01	0.27 ± 0.01	0.35 ± 0.01	0.33 ± 0.01	0.51 ± 0.01	0.21 ± 0.01	0.30 ± 0.01
Mean value difference	*p* < 0.001		*p* < 0.001		*p* < 0.001		*p* < 0.001		*p* < 0.001		*p* < 0.001	
Pull-out strength $F_{exp}$ in N	187 ± 1	209 ± 1	173 ± 2	201 ± 2	218 ± 3	248 ± 2	178 ± 2	196 ± 3	196 ± 1	221 ± 2	181 ± 2	190 ± 3
Mean value difference	*p* < 0.001		*p* < 0.001		*p* < 0.001		*p* < 0.001		*p* < 0.001		*p* < 0.001	
$D_{o} \cdot T_{i}$ in Nm·mm	1.35	2.38	1.08	2.16	2.39	3.54	1.74	2.64	2.13	3.83	1.37	2.28

^a^These screws have a double thread, therefore the thread pitch here corresponds to the distance between two thread flanks.

### Screw characteristics

**Figure 3:**
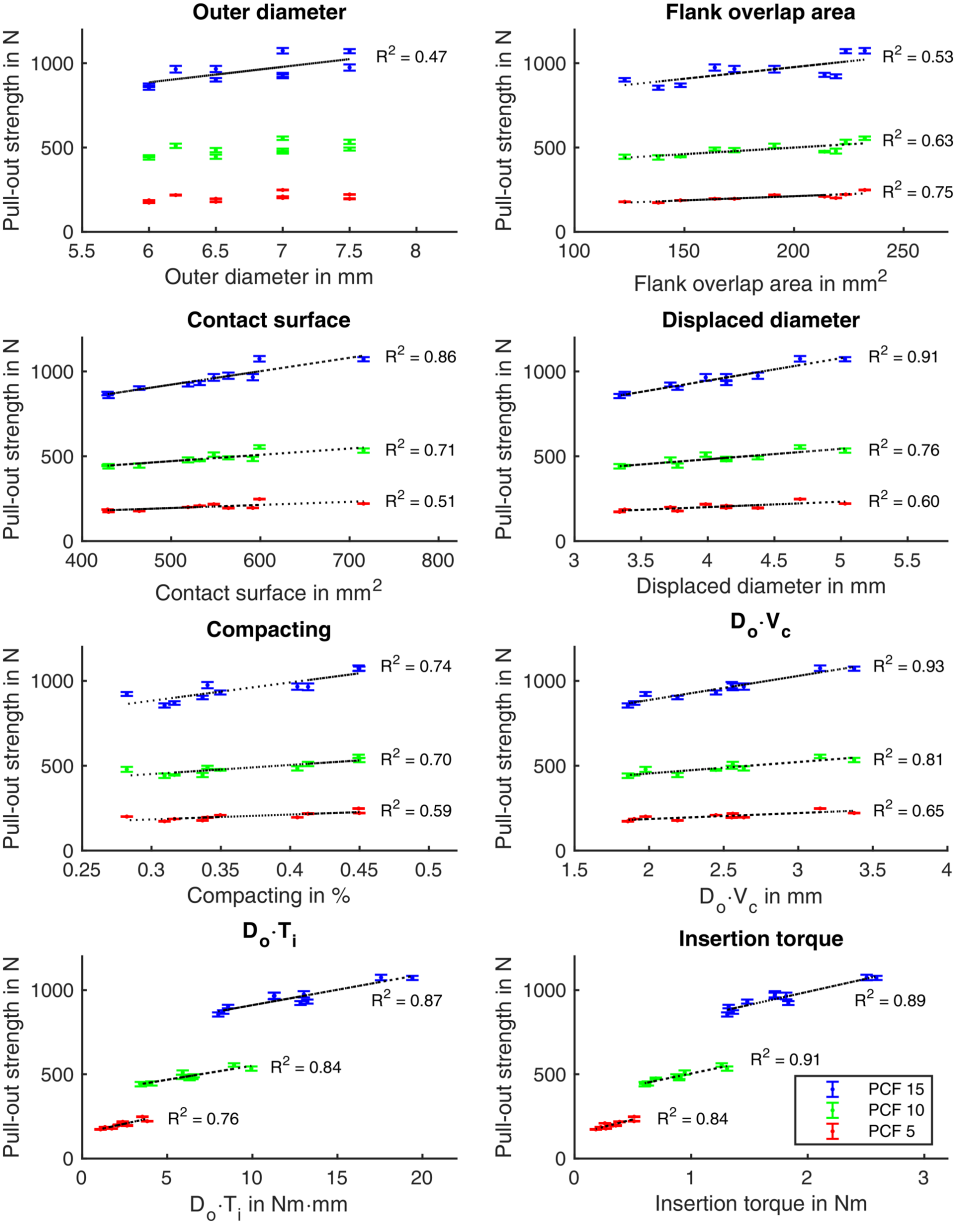
Comparison of different screw characteristics with the achieved pull-out strength for screws with tapered core. Shown are the mean values with error bars of one standard deviation for PCF 15 (blue), PCF 10 (green) and PCF 5 (red). If there was a statistically significant linear correlation, the coefficient of determination R^2^ is given.

Values for the previously defined screw characteristics can be found in Table 2. The analysis for linear regression was carried out once for screw types with tapered core (all except screw type 6) and once for all screw types. The results are shown in Table 3, with coefficients of determination R^2^ > 0.7 in italics and non-significant data highlighted in bold. The corresponding diagrams with data points of the mean values for screws with tapered core are shown in Fig. 3 and for all screw types in Appendix 2. No general correlation between the outer diameter and the pull-out strength can be found. For screws with tapered core, there is a linear relationship between FOA and the achievable pull-out strength, especially at low foam densities. The contact area clearly correlates with the pull-out strength, especially for screws with tapered core at higher foam densities. The parameters displaced diameter and compacting correlate very well with the pull-out strength for screws with tapered core. However, when all types of screws are considered, this correlation no longer exists for all densities. The insertion torque is in a strong linear relationship to the pull-out strength for all foam densities. Additionally, the term $D_{o} \cdot T_{i}$ correlates very well with the pull-out strength across all densities. In contrast, there is a strong linear correlation between the term $D_{o} \cdot V_{c}$ and the pull-out strength only in the case of screws with tapered core.

**Table 3:** Correlation results for different screw characteristics against pull-out strength for (a) screws with tapered core and (b) all screws considered.

Characteristics	$F_{P}$ for screws with tapered core (screw type 6 excluded)		
	PCF 15	PCF 10	PCF 5
**(a)**			
Outer diameter $D_{o}$	R^2^ = 0.47, p = 0.029	**R**^**2**^** = 0.35, p = 0.740**	**R**^**2**^** = 0.25, p = 0.143**
$FOA$	R^2^ = 0.53, p = 0.016	R^2^ = 0.63, p = 0.006	*R*^*2*^ = *0.75, p* < *0.001*
Contact surface $A_{c}$	*R*^*2*^ = *0.86, p* < *0.001*	*R*^*2*^ = *0.71, p* = *0.002*	R^2^ = 0.51, p = 0.020
Displaced diameter $d_{d}$	*R*^*2*^ = *0.91, p* < *0.001*	*R*^*2*^ = *0.76, p* = *0.001*	R^2^ = 0.60, p = 0.009
Compacting $V_{c}$	*R*^*2*^ = *0.74, p* = *0.001*	*R*^*2*^ = *0.70, p* = *0.002*	R^2^ = 0.59, p = 0.010
Insertion torque $T_{i}$	*R*^*2*^ = *0.89, p* < *0.001*	*R*^*2*^ = *0.91, p* < *0.001*	*R*^*2*^ = *0.84, p* < *0.001*
$D_{o} \cdot T_{i}$	*R*^*2*^ = *0.87, p* < *0.001*	*R*^*2*^ = *0.84, p* < *0.001*	*R*^*2*^ = *0.76, p* = *0.001*
$D_{o} \cdot V_{c}$	*R*^*2*^ = *0.93, p* < *0.001*	*R*^*2*^ = *0.81, p* < *0.001*	*R*^*2*^ = *0.65, p* = *0.005*

Characteristics	$F_{P}$ for all screws		
	PCF 15	PCF 10	PCF 5
**(b)**			
Outer diameter $D_{o}$	**R**^**2**^** = 0.12, p = 0.270**	**R**^**2**^** = 0.13, p = 0.257**	**R**^**2**^** = 0.16, p = 0.201**
$FOA$	**R**^**2**^** = 0.31, p = 0.062**	R^2^ = 0.45, p = 0.018	R^2^ = 0.67, p = 0.001
Contact surface $A_{c}$	R^2^ = 0.43, p = 0.021	R^2^ = 0.43, p = 0.020	R^2^ = 0.43, p = 0.022
Displaced diameter $d_{d}$	**R**^**2**^** = 0.29, p = 0.073**	**R**^**2**^** = 0.31, p = 0.060**	R^2^ = 0.39, p = 0.031
Compacting $V_{c}$	**R**^**2**^** = 0.32, p = 0.057**	R^2^ = 0.35, p = 0.041	R^2^ = 0.43, p = 0.020
Insertion torque $T_{i}$	*R*^*2*^ = *0.75, p* < *0.001*	*R*^*2*^ = *0.86, p* < *0.001*	*R*^*2*^ = *0.85, p* < *0.001*
$D_{o} \cdot T_{i}$	R^2^ = 0.65, p = 0.002	*R*^*2*^ = *0.75, p* < *0.001*	*R*^*2*^ = *0.76, p* < *0.001*
$D_{o} \cdot V_{c}$	**R**^**2**^** = 0.33, p = 0.051**	R^2^ = 0.36, p = 0.040	R^2^ = 0.44, p = 0.019

Data with high linear correlation are printed in italics, while non-significant data are shown in bold.

### Analytical evaluation

**Figure 4:**
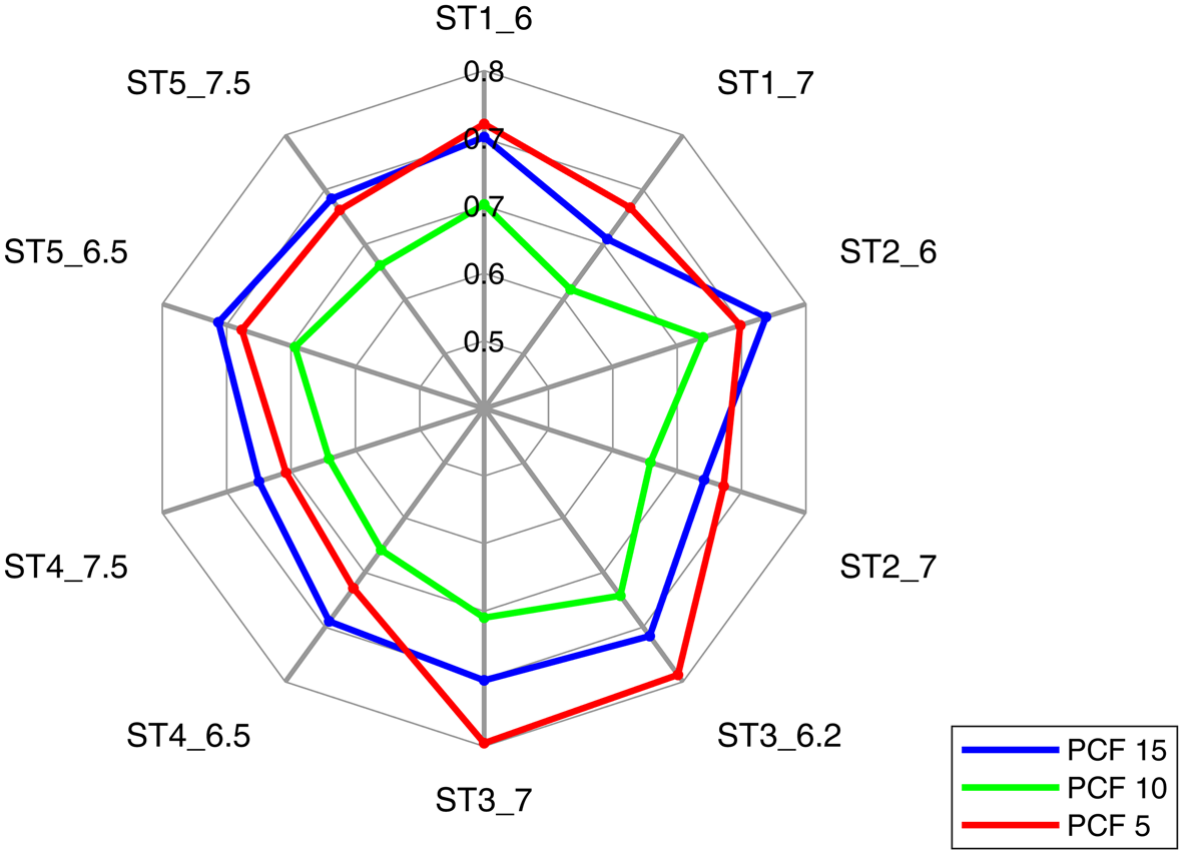
Ideal correction factors for screw pull-out determined from experimental data on different material densities.

Figure 4 gives the ideal correction factors $CF_{ideal}$ for screws with tapered core for the three material densities calculated according to Eq. (6). Each screw has a specific correction factor. Quantitatively, there is a small difference per material density. The lowest values are achieved for the medium density PCF 10. While the values for high and low density are very close, partly PCF 15 and partly PCF 5 take the highest values.

**Figure 5:**
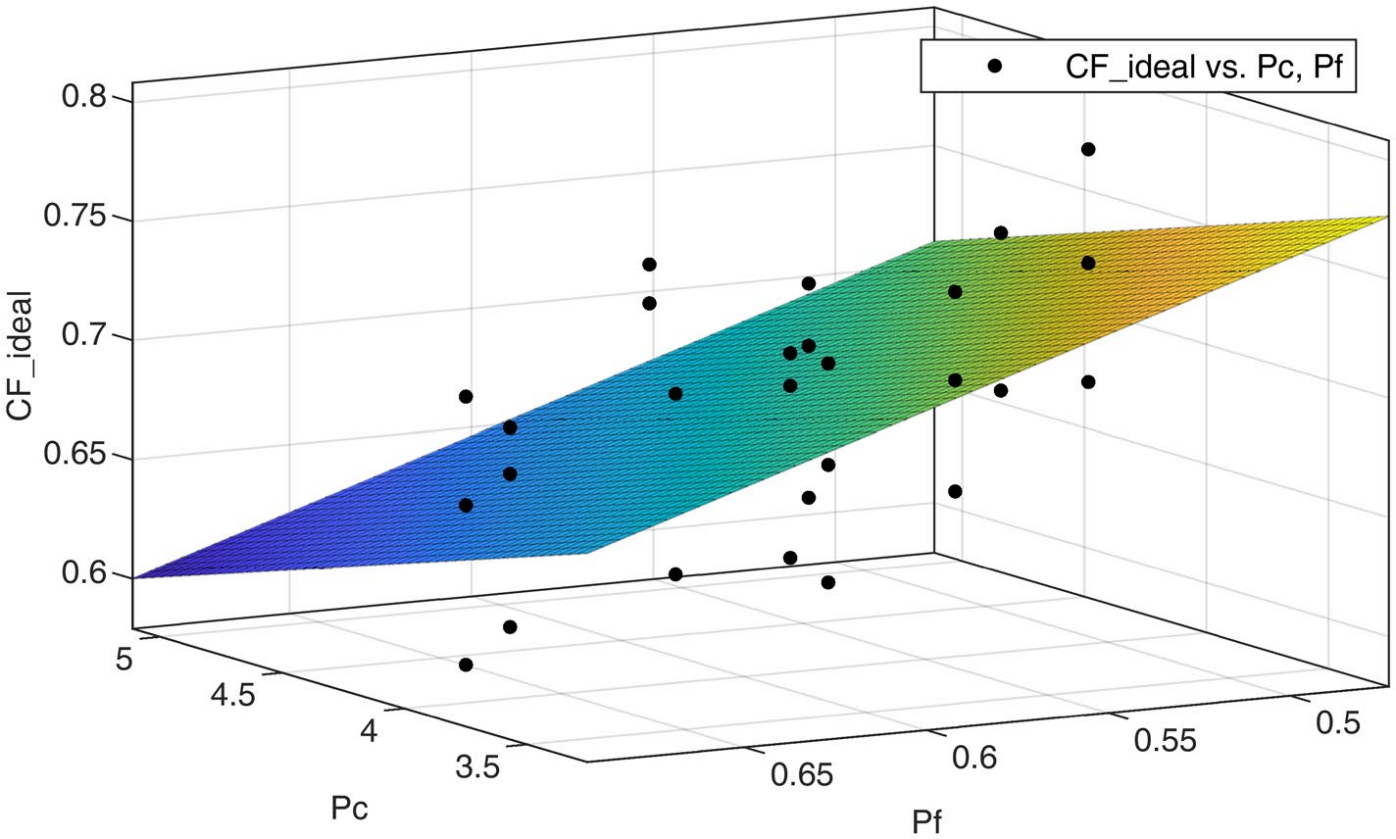
3D regression of the final selected parameters displaced diameter as P_C_ and relative flank area as P_F_ on the ideal correction factor.

**Figure 6:**
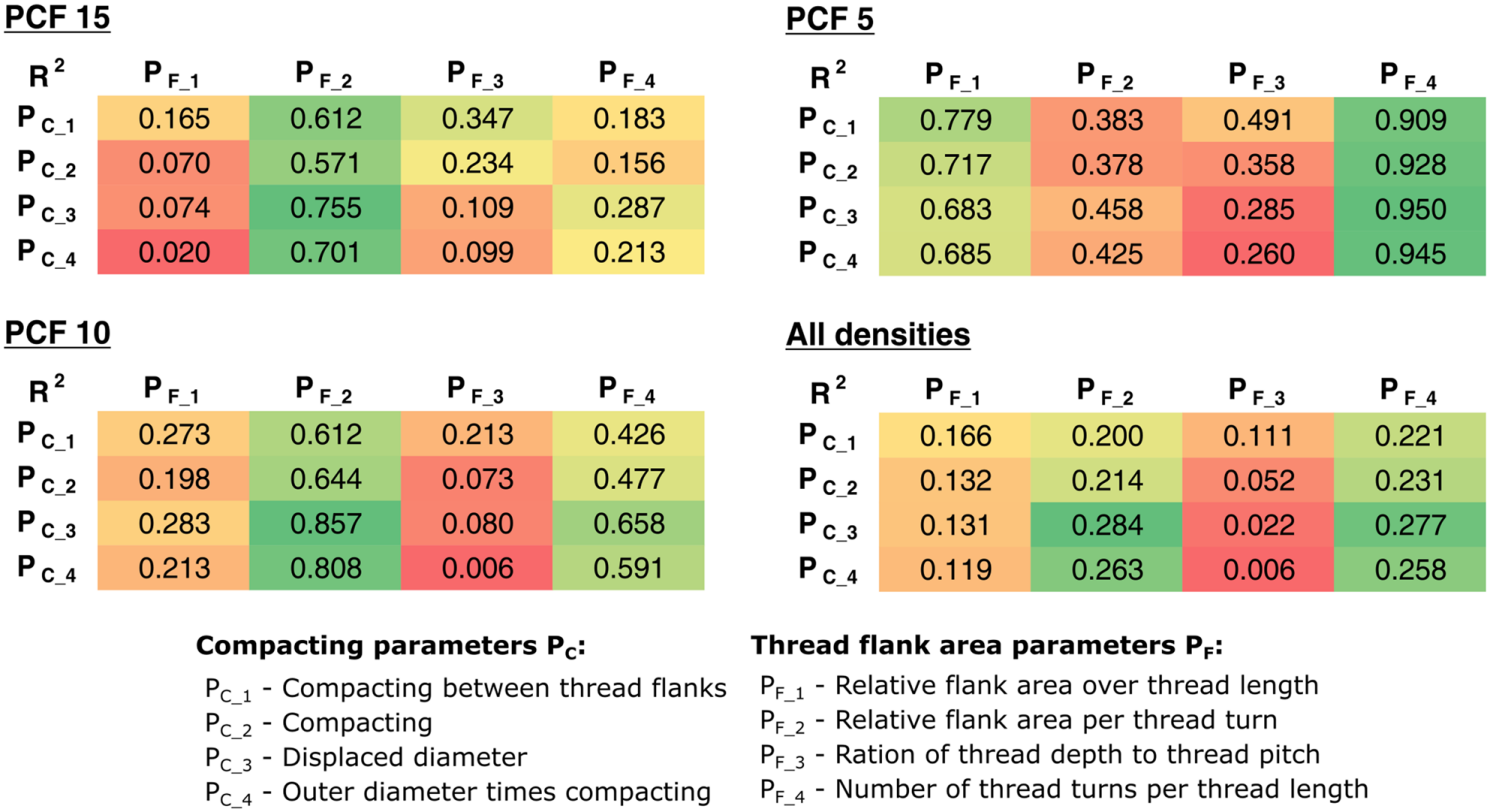
Evaluation of the analytical examination. A high coefficient of determination R^2^ indicates that the considered parameters (P_C_ and P_F_) represent the correction factor well. The most favourable combinations are highlighted in green. The comparisons were made for the individual material densities (PCF 15, PCF 10 and PCF 5) and across all material densities of the synthetic foam.

An example of a 3D regression is shown in Fig. 5. The calculated coefficient of determination R^2^ for the respective parameter combinations of $P_{C}$ and $P_{F}$ is summarised in Fig. 6. Accordingly, for the high density PCF 15 there is the best agreement for the relative flank area, especially in combination with the displaced diameter. A similar picture emerges for the medium density PCF 10, whereby the result shifts with decreasing density. For the low density PCF 5, the number of threads per thread length has the best agreement. If the parameters are fitted over the three densities, the best agreement is obtained for the displaced diameter and the relative flank area. For this parameter combination, the constants for the linear equation $CF$ were determined from Eq. (7), leading to

8 $$ CF = A \cdot P_{C\_3} + B \cdot P_{F\_2} + C = - 0.036 d_{d} \cdot \frac{1}{{\left[ {mm} \right]}} - 0.497 \frac{{D_{m}^{2} - D_{c}^{2} }}{{D_{m}^{2} }} + 1.128. $$

Based on this, the pull-out strength $F_{P}$ was calculated according to Eq. (5).

**Figure 7:**
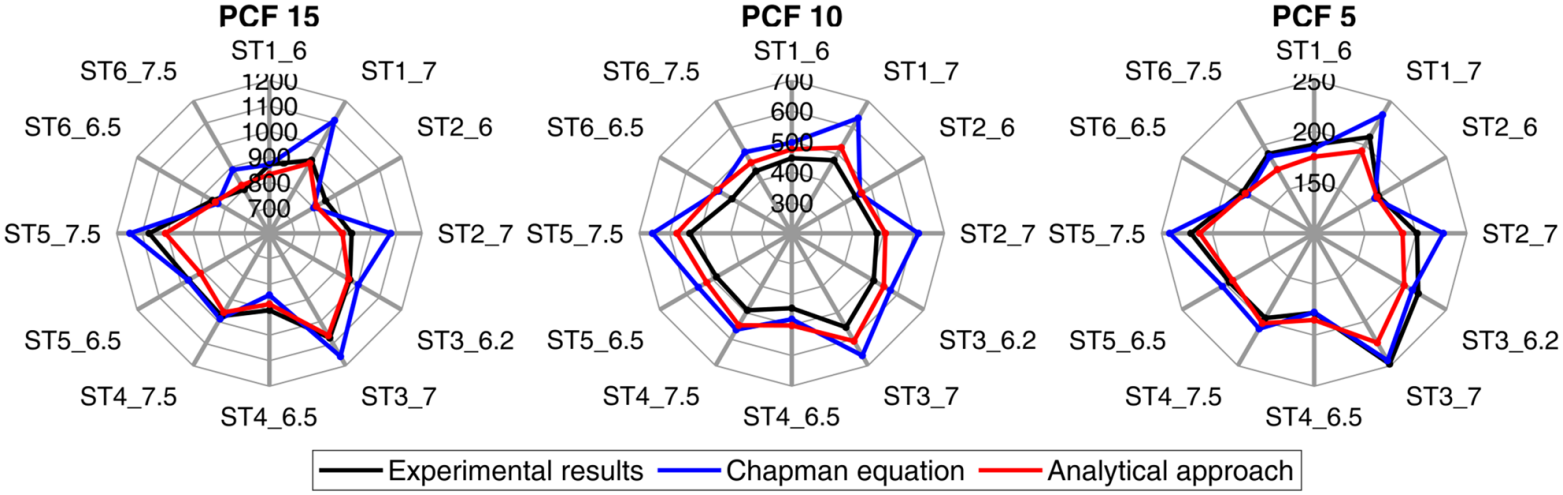
Comparison of the experimental pull-out strengths (in N) with the calculated values according to Chapman and the analytical approach for three densities of synthetic foam.

Table 5 compares the experimentally determined pull-out strength $F_{exp}$ with the analytically determined pull-out strengths. Across all densities and all screws, the Chapman equation $F_{Ch}$ gives a mean deviation of 8% (range: − 7 to 33%). In contrast, the newly introduced equation for $F_{P}$ determines the pull-out strengths with a mean deviation of 1% (range: − 10 to 13%). The pull-out strengths determined are shown for the three densities in Fig. 7.

### Donor bones

**Figure 8:**
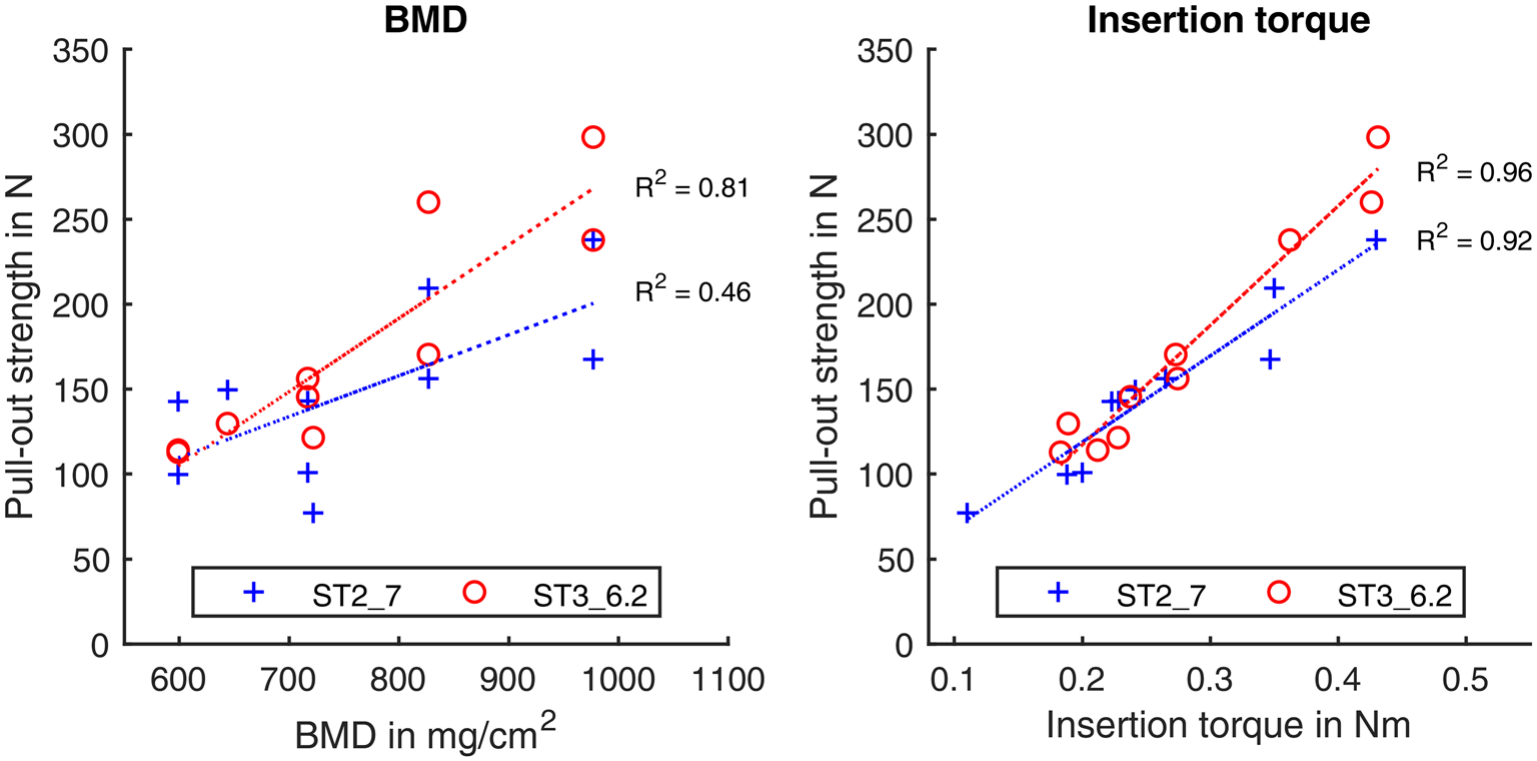
Representation of the pull-out strengths from vertebral bodies of body donors as a function of the bone mineral density BMD (left) and the insertion torque (right), with differentiation of the screws ST2_7 and ST3_6.2

The DEXA examination resulted in T-scores for one osteopenic (T-score: − 1.9) and five osteoporotic (T-score: − 3.1 to − 4.7) body donor samples. Bone mineral density ranged from 599 to 977 mg/cm^2^, cf. Table 4. The measured values for insertion torque and pull-out strength were normally distributed. The mean insertion torque determined on the donor vertebrae for ST2_7 and ST3_6.2 was 0.26 Nm (SD 0.09 Nm) and 0.28 Nm (SD 0.09 Nm), respectively. There was no significant difference (*p* = 0.18). For the pull-out strength 148 N (SD 50 N) was measured for ST2_7 and 175 N (SD 67 N) for ST3_6.2. The mean value difference in pull-out strength was 26 N (SD 35 N) and was statistically significant *p* = 0.04 (correlation coefficient: r = 0.862, 95% confidence interval of difference: 1–51 N). The Cohen's d value was determined to be 0.75, corresponding to a medium to large effect. In direct comparison, the relative mean deviation of the pull-out strength for ST3_6.2 is 12% higher than for ST2_7. A linear correlation of the pull-out strength to the BMD was found for the screw ST2_7 (R^2^ = 0.46, *p* = 0.03) and a high linear correlation for ST3_6.2 (R^2^ = 0.81, *p* < 0.001), cf. Figure 8. There was a high linear correlation between pull-out strength and insertion torque for ST2_7 (R^2^ = 0.92, *p* < 0.001) and ST3_6.2 (R^2^ = 0.96, *p* < 0.001).

**Table 4:** Data of the body donor samples.

Donor	Gender	Donor age (years)	BMD (mg/cm^2^)	T-score	Pathology
1	Female	78	599	− 4.7	
2	Female	92	717	− 4.1	
3	Female	88	644	− 4.4	th12 defect
4	Male	93	827	− 3.1	
5	Male	90	977	− 1.9	
6	Male	77	722	− 4.0	th12 defect
Mean value ± SD		86 ± 6	748 ± 137	− 3.7 ± 1.0

## Discussion

In a comprehensive study, the anchoring effect of screws in the cancellous bone was analysed. It has been shown that some screw characteristics derived from the screw design are suitable to assess the anchorage quality against pull-out from artificial cancellous bone. In fact, it seems more useful to consider two parameters together, one describing the compacting and one describing the thread flank area. To the authors' knowledge, this is the first time that the effects of two parameters in combination on the anchorage behaviour of pedicle screws have been investigated, taking into account different material densities. For screws with tapered core, the parameters of displaced diameter and relative thread flank area proved to be the most suitable. An equation derived from these parameters provides very good predictions for the expected pull-out strength and is an improvement on the Chapman equation. Furthermore, results could be verified by tests on donor material. These findings will be discussed in detail below.

Each design feature can have an influence on screw anchorage^33,34^. It is still unknown which combination of features leads to the best anchorage. The interaction of design features has hardly been considered so far. In this study, the characteristics of screws resulting from different combinations of design features were studied. These characteristics were expressed as quantitative values. As a result, comparisons are possible even for screws that differ in more than one design feature. This approach seems advantageous as different screws can be compared with each other as their thread design is described by more general characteristics.

It is often assumed that a better anchorage is achieved with a larger outer diameter^13,30,31^. In the current study, it is found for screws with tapered core that higher pull-out strengths are achieved with a larger outer diameter for one type of screw, cf. Table 2. However, a generally valid correlation between outer diameter and pull-out strength could not be established, cf. Table 3. In contrast, the screws with cylindrical core showed inconsistent behaviour at different material densities. Therefore, the outer diameter alone does not seem to be a good representative for the screw thread and thus the achievable anchorage.

For screws with tapered core, good correlations of flank overlap area FOA and contact surface to pull-out strength were found, cf. Fig. 3. However, characteristics such as outer diameter, flank overlap area, and contact surface do not take into account the screw hole preparation. But this can have a significant influence on the pull-out strength^35,36^. Therefore, these characteristics are not sufficient as stand-alone parameters for assessing screw anchorage. Krenn et al.^18^ who introduced FOA come to the same conclusion for the characteristic FOA.

The screw hole preparation influences how much material is compacted between the thread flanks. The characteristics displaced diameter ${d}_{d}$, compacting ${V}_{c}$ and ${D}_{o}\cdot {V}_{c}$ take the screw hole preparation into account and thus map the bone compacting^24^. For screws with tapered core, very good correlations to pull-out strength are achieved over these three characteristics, especially at higher foam densities, cf. Table 3. In the current study, the compacting ability seems to be more relevant than the outer diameter. The positive effect of the compacting of screws with tapered core has been described so far^19,23^. For the first time, a quantitative comparison of the compacting for a larger number of screws has been considered. This characteristic appears useful for understanding the anchoring mechanisms. However, the degree of compacting cannot be increased arbitrarily. While at higher densities the degree of compacting has a substantial influence, it seems that at lower densities FOA becomes more important, cf. Fig. 3 and Table 3. As an example, the screw ST1_7 and ST4_7.5 can be looked at. At high density PCF 15, the ST4_7.5 has a higher pull-out strength, while at the lowest density PCF 5 it is higher for the ST1_7. Both screws have comparable compacting behaviour, but the flank area of the ST1_7 is larger. Figure 4 also supports the conjecture that some screws show a deviating behaviour at lower density. There seems to be a density dependence of the anchoring mechanisms. However, there is too little data to establish a quantitative relationship. Nevertheless, this observation is highly interesting and should be verified with more material densities in the future. Furthermore, it appears that one screw characteristic to represent anchorage quality is limited. Tsai et al.^25^ come to a similar conclusion when they state that pull-out strength is the result of a number of varying parameters.

The relevance of compacting and flank area can also be observed for screws with the same outer diameter. ST3_7 has higher compacting and a larger flank area than ST1_7 and ST2_7, which is also reflected in a higher pull-out strength for all three material densities. ST1_6 and ST2_6 have similar compacting properties. However, ST1_6 has a larger flank area, which is also reflected in a higher pull-out strength. As a further result, a very good correlation between pull-out strength and insertion torque, as well as to the term ${D}_{o}\cdot {T}_{i}$, was found for all screws across all foam densities, cf. Table 3. It is often assumed that the insertion torque is a predictor for the anchorage strength^37,38^. This view is controversial in the literature and has been discussed in detail elsewhere^24^. However, the insertion torque is determined during surgery and is therefore not a characteristic that results directly from the thread design.

In an analytical evaluation, the influence of the screw design on the screw hold was mapped via two parameters as a correction factor $CF\left({P}_{C},{P}_{F}\right)$. For this purpose, different parameters for compacting ${P}_{C}$ and for thread flank area ${P}_{F}$ were introduced, and the approximation to experimentally determined ideal correction factors was evaluated. It was found that the advantageous parameter combinations change with the density of the test material, cf. Fig. 6. For the high density PCF 15, compacting and relative flank area ${P}_{F\_2}$ showed the best correlation. Accordingly, the number of thread turns seems to be less important. At low density PCF 5, good agreement is achieved with compacting and relative flank area over thread length ${P}_{F\_1}$. This is a dimensionless measure of FOA and includes the number of thread turns. Even better agreement is achieved by combining compacting and number of thread turns per thread length ${P}_{F\_4}$ for the lowest density. Presumably, at very low densities, it is not a large flank area but a high number of engaging thread flanks that has a greater influence on the anchorage. In other studies it was found that with a decreased thread pitch (i.e. more thread flanks in engagement) better anchorage is achieved^14,39,40^. Asnis et al.^39^ mentioned that this effect is more pronounced at low densities. In the current study, different compacting parameters are considered, but it seems that compacting is best represented by the displaced diameter.

For screws with tapered core, an equation for calculating the pull-out strength from the parameters displaced diameter and relative flank area was presented, cf. Equation (5) with Eq. (8). The results are close to the experimental measurements, also for screw type 6, cf. Table 5. The Chapman equation, on the other hand, shows clearly greater deviations in some cases. This may be the case because the effects of pre-drilling or tapping and the compressive effect of modern screws are not taken into account^25^. In Table 5 it is noticeable that the analytical approach underestimates the pull-out strengths for high density, overestimates them for medium density and underestimates them for low density. This is probably due to the fact that tabulated values were used for the shear forces and the real material properties differ from these. For polyurethane foams, there is a sensitive relationship between the shear force and the apparent density^14,39^. In addition, the difference between the ideal correction factor ${CF}_{ideal}$ of one screw at different densities, shown in Fig. 4, can be partly explained by deviating material properties. Tsai et al.^25^ presented a complex integral approach for the calculation of the pull-out strength from the screw design. They achieved a mean deviation of − 5% (range: − 10 to − 2%). For this, they had determined the shear strength of the synthetic test material and used only one density. It stands to reason that with the approach presented, the pull-out strengths could be calculated even more accurately if only one density is considered. This can be seen from the good correlations of the individual densities, cf. Fig. 6.

**Table 5:** Comparison of the analytical calculations with the measurement results.

	Screw type 1		Screw type 2		Screw type 3		Screw type 4		Screw type 5		Screw type 6	
	ST1_6	ST1_7	ST2_6	ST2_7	ST3_6.2	ST3_7	ST4_6.5	ST4_7.5	ST5_6.5	ST5_7.5	ST6_6.5	ST6_7.5
**Measured results**												
*PCF 15* $\tau_{S} =\,$ $2.8 \;{\text{MPa}}$												
Measured pull-out strength $F_{exp}$ in N^a^	870 ± 10	931 ± 12	855 ± 12	923 ± 12	965 ± 20	1074 ± 16	902 ± 10	975 ± 19	966 ± 18	1072 ± 12	858 ± 19	798 ± 24
Chapman pull-out strength $F_{Ch}$ in N	870	1112	801	1076	1003	1158	842	987	968	1148	834	888
Deviation to $F_{exp}$	0%	19%	− 6%	17%	4%	8%	− 7%	1%	0%	7%	− 3%	11%
Analytical pull-out strength $$ F_{P}$$ in N	831	917	813	887	959	1063	878	958	913	1008	845	817
Deviation to $F_{exp}$	− 4%	− 2%	− 5%	− 4%	− 1%	− 1%	− 3%	− 2%	− 5%	− 6%	− 2%	2%
*PCF 10 *$\tau_{S} =\,$ $1.6 \;{\text{MPa}}$												
Measured pull-out strength $F_{exp}$ in N^a^	446 ± 4	476 ± 5	441 ± 13	479 ± 15	510 ± 13	555 ± 10	445 ± 12	491 ± 9	485 ± 13	534 ± 13	426 ± 6	435 ± 17
Chapman pull-out strength $F_{Ch}$ in N	497	635	458	615	573	662	481	564	553	656	476	507
Deviation to $F_{exp}$	11%	33%	4%	28%	12%	19%	8%	15%	14%	23%	12%	17%
Analytical pull-out strength $F_{P}$ in N	475	524	465	507	548	607	502	548	522	576	483	467
Deviation to $F_{exp}$	6%	10%	5%	6%	7%	9%	13%	12%	8%	8%	13%	7%
PCF 5 $\tau_{S} =\,$ $0.59\;{\text{ MPa}}$												
Measured pull-out strength $F_{exp}$ in N^a^	187 ± 1	209 ± 1	173 ± 2	201 ± 2	218 ± 3	248 ± 2	178 ± 2	196 ± 3	196 ± 1	221 ± 2	181 ± 2	190 ± 3
Chapman pull-out strength $F_{Ch}$ in N	183	234	169	227	211	244	177	208	204	242	176	187
Deviation to $F_{exp}$	− 2%	12%	− 2%	13%	− 3%	− 2%	0%	6%	4%	9%	− 3%	− 2%
Analytical pull-out strength $F_{P}$ in N	175	193	171	187	202	224	185	202	192	212	178	172
Deviation to $F_{exp}$	− 6%	− 8%	− 1%	− 7%	− 7%	− 10%	4%	3%	− 2%	− 4%	− 2%	− 9%

^a^Given as mean value ± one standard deviation.

In any case, it was not the intention of this study to derive a new universal equation for calculating the pull-out strength of pedicle screws. This would require further research to map, among other things, the compacting for different screw hole preparation techniques and the relationship with changing material density. The aim was rather to verify the assumption that the anchoring effect against pull-out is determined by the compacting and the thread flank area. In Eq. (8), the parameters displaced diameter and relative flank area were found to be most suitable. The calculated forces are in very good agreement with the experimental data. This proves the relevance of the parameters considered and sustains the assumption.

Furthermore, the aim was to verify the findings from the tests on synthetic foam on donor material as well. For this purpose, the screws ST2_7 and ST3_6.2 were considered. Due to the larger outer diameter, the higher FOA and the higher d/p ratio, a higher pull-out strength could be expected for ST2_7 than for ST3_6.2^13,14,18,39,40^. However, a significantly higher pull-out strength in the donor material was demonstrated for ST3_6.2, as had already been done for synthetic foam. Consequently, the screw characteristics of ST3_6.2 result in a stronger screw-bone bond compared to ST2_7. The screw design had an effect on the anchorage. Presumably, the higher compacting effect of this screw leads to better anchorage. Possibly, this is further enhanced in low bone quality due to the larger relative thread flank area. In any case, this is an interesting result and should be further investigated in subsequent studies with more screws and specimens. Furthermore, as in other studies with donor material, a correlation between BMD and pull-out strength as well as between insertion torque and pull-out strength (cf. Fig. 8) was found^2,37,41^.

The findings from this study can be used to improve screw designs in the future. For this purpose, screws with adapted designs can be manufactured and tested; this procedure has already been successfully applied in the literature^15–17^. To reduce the experimental effort, validated finite element models can also be used^12,33,42^.

The used model of screw pull-out from a synthetic vertebral body replacement does not directly correspond to the clinical situation and needs to be discussed. Screw pull-out is not the most critical failure mode for pedicle screws. Recent studies report screw loosening with a prevalence of 15.2–40.4%^43,44^. The prevalence of screw pull-out, on the other hand, is reported to be 7.8–16.2%^43–45^. However, screw pull-out is considered a risk factor for screw loosening^43^. Thus, it has clinical relevance. Furthermore, pull-out tests are well established as an initial assessment of pedicle screws, with higher pull-out strength often associated with better anchorage^3,19,41^. Polyurethane foam has shown similar material behaviour to human trabecular bone in compression tests^46,47^. Thus, consistent results were also found between artificial and biological test material in pull-out tests^22,37,48^. Therefore, polyurethane foam is considered a suitable substitute material for cancellous bone in quasi-static tests. In contrast, polyurethane foam shows brittle material behaviour in fracture tests and may not be suitable for this type of testing^49^. A major advantage is that fewer specimens are required with polyurethane foam compared to tests with bone tissue^14^. In the current study, this is shown by a low scatter of the measured values for polyurethane foam, cf. Table 2. In addition, solely the anchorage in the region of the vertebral body was considered. In the literature, pull-out tests are often carried out over the entire length of the pedicle screw in synthetic foam. This approach did not seem to be appropriate for the intention of the present study, as different effects and influences are mixed. Biomechanically, the anchorage in the vertebra consists of two parts, the one in the vertebral body and the one in the pedicle canal^48,50^. Both areas have different structures and thus different properties. Some pedicle screws have a two-part design to meet these requirements^22,51,52^. If the entire length of the screw is tested in a uniform material, the effect of the individual thread sections can no longer be distinguished. Furthermore, the thread seems to be particularly relevant for anchorage in cancellous bone^48^. The pedicle canal is cortically characterised, so other mechanisms may influence anchorage^48,50,53^. One aim of this study was to examine the interactions between thread design and cancellous bone. Therefore, the focus of the present study is on screw anchorage in the vertebral body. However, about 60% of the screw pull-out strength is caused by the pedicle^50,54,55^. As only the anchorage in the vertebral body was considered in this study, no clinical conclusions can be drawn. Nevertheless, as the vertebral body has a significant influence on the anchorage of pedicle screws, the results are of clinical interest^50,55^.

The present study has limitations. The fact that the findings only refer to the cancellous area of the vertebral body has already been discussed in detail. An extension to the entire vertebra and further load cases should be the task of subsequent studies. The compacting effect and the thread flank area are often not independent of each other. With the same pitch, higher compression is achieved with increasing core diameter, but also lower flank area. Presumably, depending on the material density, there is an optimum between compacting and necessary thread flank area. The equation introduced [Eq. (5) with Eq. (8)] does not fulfil a general validity but is based on the measurement results of the examined screws. However, the potential was shown that the pull-out strength can be calculated from more general screw characteristics. Furthermore, the tests with body donor material were only carried out on ten specimens. Due to inter-individual differences, larger standard deviations occurred. Nevertheless, a significant difference between the compared screws could be demonstrated. Furthermore, the tests were only carried out on donor material for two of the twelve screws. Further tests on donor material could further validate the findings. The slightly different test set-up and dimensions of the specimens regarding polyurethane foam and bone can also have little influence on the results. However, it has been shown that the results obtained with synthetic foam can also be verified with donor material. In addition, the limited availability and the large number of specimens required is generally a limiting factor in body donor studies.

## Conclusion

In order to increase the anchorage of a screw in the cancellous bone against pull-out, a larger outer diameter of the same screw type can be chosen. Alternatively, the anchoring effect can be further increased by choosing an appropriate screw design. It has been shown that the anchoring effect in cancellous bone can be well described by the combination of two characteristics, one of which is the bone compacting and one of which is the thread flank area. The influence of the two characteristics is density dependent. If the bone quality is sufficient, screws with a high compaction effect are advantageous, whereas if the bone density is low, a high thread flank area (e.g. low thread pitch) also appears necessary for better screw anchorage.

## Supplementary Information

Supplementary Information.

## References

[1] Seller K (2007). Pullout strength of anterior spinal instrumentation: A product comparison of seven screws in calf vertebral bodies. Eur. Spine J. Off. Publ. Eur. Spine Soc. Eur. Spinal Deform. Soc. Eur. Sect. Cerv. Spine Res. Soc..

[2] Soshi S, Shiba R, Kondo H, Murota K (1991). An experimental study on transpedicular screw fixation in relation to osteoporosis of the lumbar spine. Spine.

[3] Kim Y-Y, Choi W-S, Rhyu K-W (2012). Assessment of pedicle screw pullout strength based on various screw designs and bone densities-an ex vivo biomechanical study. Spine J. Off. J. N. Am. Spine Soc..

[4] Shea TM (2014). Designs and techniques that improve the pullout strength of pedicle screws in osteoporotic vertebrae: current status. Biomed. Res. Int..

[5] Varghese V, Ramu P, Krishnan V, Saravana Kumar G (2016). Pull out strength calculator for pedicle screws using a surrogate ensemble approach. Comput. Methods Programs Biomed..

[6] Krag MH (1986). An internal fixator for posterior application to short segments of the thoracic, lumbar, or lumbosacral spine. Design and testing. Clin. Orthop. Relat. Res..

[7] Karami KJ (2015). Biomechanical evaluation of the pedicle screw insertion depth effect on screw stability under cyclic loading and subsequent pullout. J. Spinal Disord. Tech..

[8] Misenhimer GR, Peek RD, Wiltse LL, Rothman SL, Widell EH (1989). Anatomic analysis of pedicle cortical and cancellous diameter as related to screw size. Spine.

[9] Santoni BG (2009). Cortical bone trajectory for lumbar pedicle screws. Spine J. Off. J. N. Am. Spine Soc..

[10] Zindrick MR (1987). Analysis of the morphometric characteristics of the thoracic and lumbar pedicles. Spine.

[11] Morgan EF, Unnikrisnan GU, Hussein AI (2018). Bone mechanical properties in healthy and diseased states. Annu. Rev. Biomed. Eng..

[12] Hsu C-C (2005). Increase of pullout strength of spinal pedicle screws with conical core. Biomechanical tests and finite element analyses. J. Orthop. Res. Off. Publ. Orthop. Res. Soc..

[13] Skinner R, Maybee J, Transfeldt E, Venter R, Chalmers W (1990). Experimental pullout testing and comparison of variables in transpedicular screw fixation. A biomechanical study. Spine.

[14] Chapman JR (1996). Factors affecting the pullout strength of cancellous bone screws. J. Biomech. Eng..

[15] Karakaşlı A, Acar N, Hüsemoğlu RB (2021). Biomechanical comparison of pullout strengths of six pedicle screws with different thread designs. Joint Dis. Relat. Surg..

[16] Lee E (2019). Experimental evaluation of screw pullout force and adjacent bone damage according to pedicle screw design parameters in normal and osteoporotic bones. Appl. Sci..

[17] Liu M-Y (2020). Biomechanical comparison of pedicle screw fixation strength in synthetic bones: Effects of screw shape, core/thread profile and cement augmentation. PLoS ONE.

[18] Krenn MH, Piotrowski WP, Penzkofer R, Augat P (2008). Influence of thread design on pedicle screw fixation. Laboratory investigation. J. Neurosurg. Spine.

[19] Abshire BB, McLain RF, Valdevit A, Kambic HE (2001). Characteristics of pullout failure in conical and cylindrical pedicle screws after full insertion and back-out. Spine J. Off. J. N. Am. Spine Soc..

[20] Inceoglu S, Ferrara L, McLain RF (2004). Pedicle screw fixation strength. Pullout versus insertional torque. Spine J. Off. J. N. Am. Spine Soc..

[21] Zdeblick TA, Kunz DN, Cooke ME, McCabe R (1993). Pedicle screw pullout strength. Correlation with insertional torque. Spine.

[22] Yaman O (2015). The comparison of pullout strengths of various pedicle screw designs on synthetic foams and ovine vertebrae. Turk. Neurosurg..

[23] Amaritsakul Y, Chao C-K, Lin J (2014). Comparison study of the pullout strength of conventional spinal pedicle screws and a novel design in full and backed-out insertions using mechanical tests. Proc. Inst. Mech. Eng. Part H J. Eng. Med..

[24] Weidling M, Oefner C, Schoenfelder S, Heyde C-E (2020). A novel parameter for the prediction of pedicle screw fixation in cancellous bone—a biomechanical study on synthetic foam. Med. Eng. Phys..

[25] Tsai W-C (2009). Comparison and prediction of pullout strength of conical and cylindrical pedicle screws within synthetic bone. BMC Musculoskelet. Disord..

[26] ASTM F1839-08. *Standard Specification for Rigid Polyurethane Foam for Use as a Standard Material for Testing Orthopaedic Devices and Instruments* (ASTM International, West Conshohocken, PA, 2008).

[27] Weidling M, Wendler T, Schoenfelder S, Heyde C-E (2022). Recommendations for standardised screw pull-out from polyurethane foam—the influence of density variations of the test foam and the insertion method. Med. Eng. Phys..

[28] ASTM D1622-08. *Standard Test Method for Apparent Density of Rigid Cellular Plastics* (ASTM International, West Conshohocken, PA, 2008).

[29] ASTM F543-13. *Standard Specification and Test Methods for Metallic Medical Bone Screws* (ASTM International, West Conshohocken, PA, 2013).

[30] Bianco R-J, Arnoux P-J, Wagnac E, Mac-Thiong J-M, Aubin C-É (2017). Minimizing pedicle screw pullout risks: A detailed biomechanical analysis of screw design and placement. Clin. Spine Surg..

[31] Cho W, Cho SK, Wu C (2010). The biomechanics of pedicle screw-based instrumentation. J. Bone Joint Surg. Br..

[32] Cohen J (1988). Statistical Power Analysis for the Behavioral Sciences.

[33] Chatzistergos PE, Magnissalis EA, Kourkoulis SK (2010). A parametric study of cylindrical pedicle screw design implications on the pullout performance using an experimentally validated finite-element model. Med. Eng. Phys..

[34] Takenaka S (2020). Influence of novel design alteration of pedicle screw on pull-out strength: A finite element study. J. Orthop. Sci. Off. J. Jpn. Orthop. Assoc..

[35] Carmouche JJ, Molinari RW, Gerlinger T, Devine J, Patience T (2005). Effects of pilot hole preparation technique on pedicle screw fixation in different regions of the osteoporotic thoracic and lumbar spine. J. Neurosurg. Spine.

[36] Pfeiffer FM, Abernathie DL, Smith DE (2006). A comparison of pullout strength for pedicle screws of different designs. A study using tapped and untapped pilot holes. Spine.

[37] Daftari TK, Horton WC, Hutton WC (1994). Correlations between screw hole preparation, torque of insertion, and pullout strength for spinal screws. J. Spinal Disord..

[38] Okuyama K (2000). Can insertional torque predict screw loosening and related failures? An in vivo study of pedicle screw fixation augmenting posterior lumbar interbody fusion. Spine.

[39] Asnis SE (1996). Cancellous bone screw thread design and holding power. J. Orthop. Trauma.

[40] DeCoster TA, Heetderks DB, Downey DJ, Ferries JS, Jones W (1990). Optimizing bone screw pullout force. J. Orthop. Trauma.

[41] Pfeiffer M (1996). Effect of specimen fixation method on pullout tests of pedicle screws. Spine.

[42] Varghese, V., Kumar, G. S. & Venkatesh, K. A finite element analysis based sensitivity studies on pull out strength of pedicle screw in synthetic osteoporotic bone models. In *2016 IEEE EMBS Conference on Biomedical Engineering and Sciences (IECBES)* (IEEE 2016), pp. 382–387.

[43] Ohba T, Ebata S, Oba H, Koyama K, Haro H (2019). Risk factors for clinically relevant loosening of percutaneous pedicle screws. Spine Surg. Relat. Res..

[44] Marie-Hardy L, Pascal-Moussellard H, Barnaba A, Bonaccorsi R, Scemama C (2020). Screw loosening in posterior spine fusion: Prevalence and risk factors. Glob. Spine J..

[45] Sumiya S (2021). Comparative analysis of clinical factors associated with pedicle screw pull-out during or immediately after surgery between intraoperative cone-beam computed tomography and postoperative computed tomography. BMC Musculoskelet. Disord..

[46] Szivek JA, Thomas M, Benjamin JB (1993). Characterization of a synthetic foam as a model for human cancellous bone. J. Appl. Biomater. Off. J. Soc. Biomater..

[47] Szivek JA, Thompson JD, Benjamin JB (1995). Characterization of three formulations of a synthetic foam as models for a range of human cancellous bone types. J. Appl. Biomater. Off. J. Soc. Biomater..

[48] Tsuang F-Y (2016). Biomechanical arrangement of threaded and unthreaded portions providing holding power of transpedicular screw fixation. Clin. Biomech. (Bristol, Avon).

[49] Bokam P (2021). Fracture behavior of cancellous bone and cancellous bone-PMMA bone cement interface: An experimental study using an integrated methodology (wedge splitting test and Heaviside-based digital image correlation). J. Mech. Behav. Biomed. Mater..

[50] Hirano T (1997). Structural characteristics of the pedicle and its role in screw stability. Spine.

[51] Brasiliense LBC (2013). Characteristics of immediate and fatigue strength of a dual-threaded pedicle screw in cadaveric spines. Spine J. Off. J. N. Am. Spine Soc..

[52] Wiendieck K, Müller H, Buchfelder M, Sommer B (2018). Mechanical stability of a novel screw design after repeated insertion: can the double-thread screw serve as a back up?. J. Neurosurg. Sci..

[53] Albanese K, Ordway NR, Albanese SA, Lavelle WF (2017). Effect of pedicle fill on axial pullout strength in spinal fixation after rod reduction. Orthopedics.

[54] Weinstein JN, Rydevik BL, Rauschning W (1992). Anatomic and technical considerations of pedicle screw fixation. Clin. Orthop. Relat. Res..

[55] Hsieh M-K (2020). Use of longer sized screws is a salvage method for broken pedicles in osteoporotic vertebrae. Sci. Rep..

