# Supplementary material for: Influence of thread design on anchorage of pedicle screws in cancellous bone: an experimental and analytical analysis
Source: Sci Rep. 2022 May 16;12:8051. doi: 10.1038/s41598-022-11824-2 (PMC9110386; doi:10.1038/s41598-022-11824-2)
Supplement: Supplementary file 1 — Supplementary Information. [file 41598_2022_11824_MOESM1_ESM.pdf]

Appendix

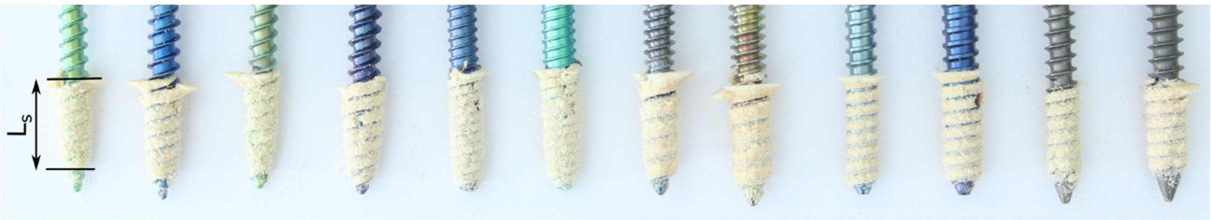

Appendix 1: Determination of the shear length L_s.

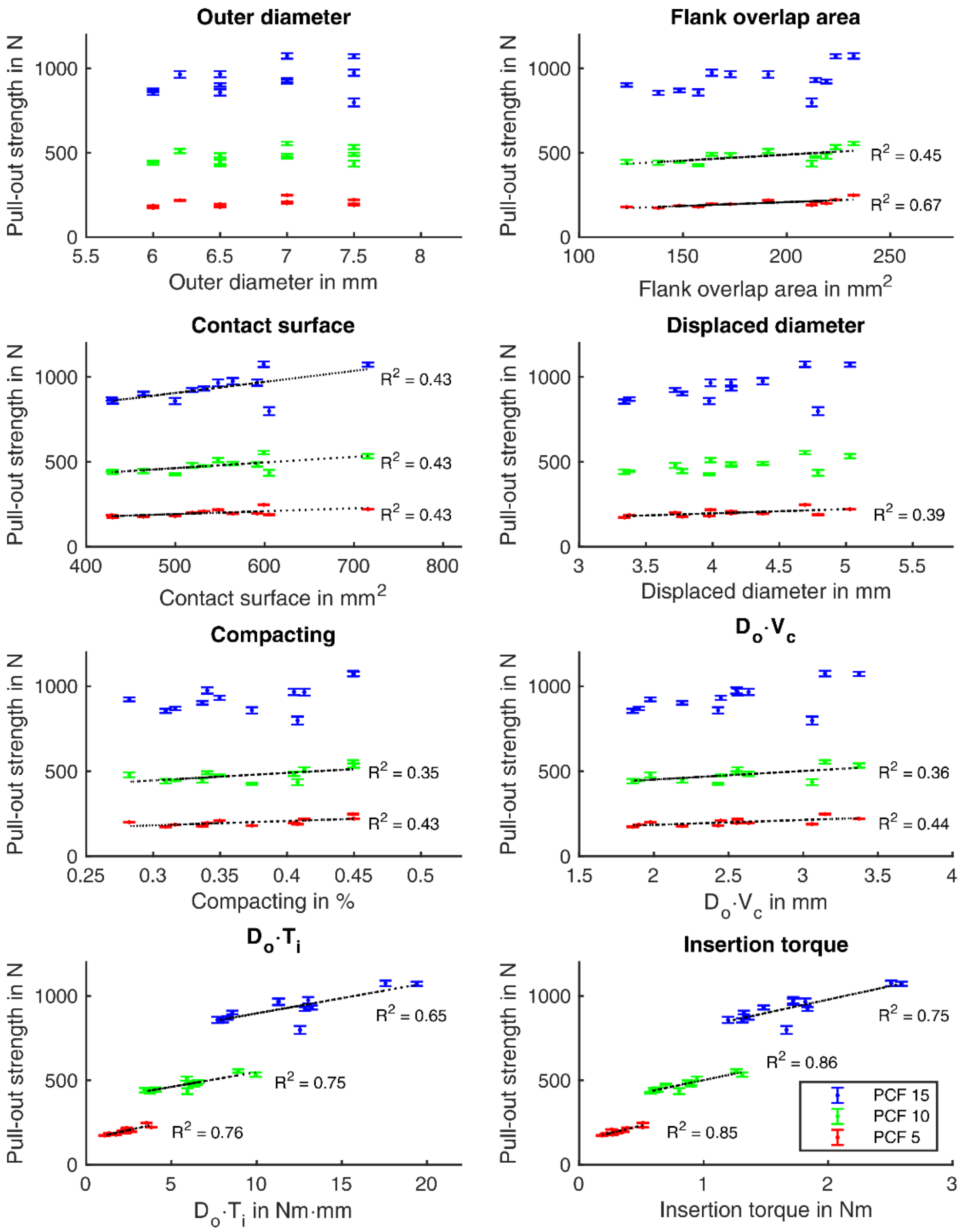

Appendix 2: Comparison of different screw characteristics with the achieved pull-out strength for all considered screws. Shown are the mean values with error bars of one standard deviation for PCF 15 (blue), PCF 10 (green) and PCF 5 (red). If there was a statistically significant linear correlation, the coefficient of determination R^2 is given.
